# Microevolution of *Bartonella grahamii* driven by geographic and host factors

**DOI:** 10.1128/msystems.01089-24

**Published:** 2024-09-30

**Authors:** Ailing Xu, Liang Lu, Wen Zhang, Xiuping Song, Guichang Li, Yu Miao, Ruixiao Li, Min Chen, Qiyong Liu, Dongmei Li

**Affiliations:** 1 National Key Laboratory of Intelligent Tracking and Forecasting for Infectious Diseases, Department of Vector Biology and Control, National Institute for Communicable Disease Control and Prevention, Chinese Center for Disease Control and Prevention, Beijing, People's Republic of China; University of California San Diego, La Jolla, California, USA

**Keywords:** *Bartonella grahamii*, population structure, comparative genomics, phylogenomic analysis, phylogeography

## Abstract

*Bartonella grahamii* is one of the most prevalent *Bartonella* species in wild rodents and has been associated with human cases of neuroretinitis. The structure and distribution of genomic diversity in natural *B. grahamii* is largely unexplored. Here, we have applied a comprehensive population genomic and phylogenomic analysis to 172 strains of *B. grahamii* to unravel the genetic differences and influencing factors that shape its populations. The findings reveal a remarkable genomic diversity within the species, primarily in the form of single- nucleotide polymorphisms. The open pangenome of *B. grahamii* indicates a dynamic genomic evolution influenced by its ecological niche. Whole-genome data allowed us to decompose *B. grahamii* diversity into six phylogroups, each characterized by a unique “mosaic pattern” of hosts and biogeographic regions. This suggests a complex interplay between host specificity and biogeography. In addition, our study suggests a possible origin of European strains from Asian lineages, and host factors have a more significant impact on the genetic differentiation of *B. grahamii* than geographical factors. These insights contribute to understanding the evolutionary history of this pathogen and provide a foundation for future epidemiological research and public health strategies.

**IMPORTANCE:**

*Bartonella grahamii* has been reported worldwide and shown to infect humans. Up to now, an effective transmission route of *B. grahamii* to humans has not been confirmed. The genetic evolution of *B. grahamii* and the relationship between *B. grahamii* and its host need to be further studied. The factors driving the genetic diversity of *B. grahamii* are still controversial. The results showed that the European isolates shared a common ancestor with the Chinese isolates. Host factors were shown to play an important role in driving the genetic diversity of *B. grahamii*. When host factors were fixed, geographic barriers drove *B. grahamii* microevolution. Our study emphasizes the importance of characterizing isolate genomes derived from hosts and geographical locations and provides a new reference for the origin of *B. grahamii*.

## INTRODUCTION

*Bartonella grahamii* (*B. grahamii*) was first identified in the blood of moles in 1905, isolated from blood of *Myodes glareolus* in the United Kingdom and assigned *Grahamella* to the genus *Bartonella* (1–3). So far, *B. grahamii* has been reported in 31 countries around the world, including China (4). In China, *B. grahamii* is the dominant species and wide distributed (5) covering 21 provinces (municipalities or autonomous regions) and 7 biogeographic regions (6).

Currently, rodents are the main host animals of *B. grahamii*, a total of 22 genera and about 42 species, including Muridae, Cricetidae, Sciuridae, Dipodidae, and Myoxidae. Notably, *B. grahamii* has been detected in *Sciurus vulgaris*, *Erinaceus europaeus*, *Erinaceus romanicus* (7), and *Hydropotes inermis argyropus* (8) in recent years. This suggests that these animals may serve as new hosts. Studies have shown that *B. grahamii* infects humans and causes serious ophthalmic complications (9). Fleas and ticks play a crucial role in the transmission of *B. grahamii* among rodents (10). Blood-sucking arthropods are the main vectors for the transmission of *B. grahamii* in rodents. The *Haemadipsa rjukjuana*, which sucks the blood of humans and animals, has been reported to also carry *B. grahamii* (11). This suggests that members of the Annelida may also serve as vectors for the transmission of *B. grahamii*. Yet, the effective transmission route of *B. grahamii* to humans remains to be confirmed. The genetic evolution of *B. grahamii* and the correlation between *B. grahamii* and host await for being further elucidated.

Although *B. grahamii* has been reported in many countries worldwide, limited studies on its genetic diversity and population structure were conducted. Strong geographic clustering of *B. grahamii* from Asia, Europe, and North America was found (12, 13). However, based on the concatenated sequence analysis of 16S rRNA, *ftsZ*, *gltA,* and *rpoB* genes and the diversity of *gltA* genes, we did not find obvious geographical clustering strains from Asia, Europe, and North America, instead the strains were clustered by host species (14). Analysis of population structure and origin with gene fragments of *B. grahamii* tends to be inconclusive. To this point, we analyzed the population structure and phylogenetic relationships of *B. grahamii* at genomic level to evaluate the contribution of host and geographical distribution on its diversity.

## RESULTS

### Phylogenetic relationships of global *B. grahamii*

Based on the *gltA* gene screening, a total of 167 *B. grahamii* strains isolated from the wild in China were included in this study. One hundred and sixty-seven strains were isolated from 25 sampling sites in 6 biogeographic regions. Fifty-six (33.53%), 32 (19.16%), 31 (18.56%), 14 (8.38%), 9 (5.39%), and 25 (14.97%) *B. grahamii* strains were collected from Qinghai-Tibet, North China, Northeast, Inner Mongolia-Xinjiang, Central China, and Southwest biogeographic regions in China, respectively. Qinghai-Tibet, North China, Northeast and Inner Mongolia-Xinjiang areas belong to Palaearctic region, and Central China and Southwest areas belong to oriental region. All *B. grahamii* isolates were established from tissue samples of rodents belonging to 23 species, 13 genera, 5 families (Table S1).

**Fig 1:**
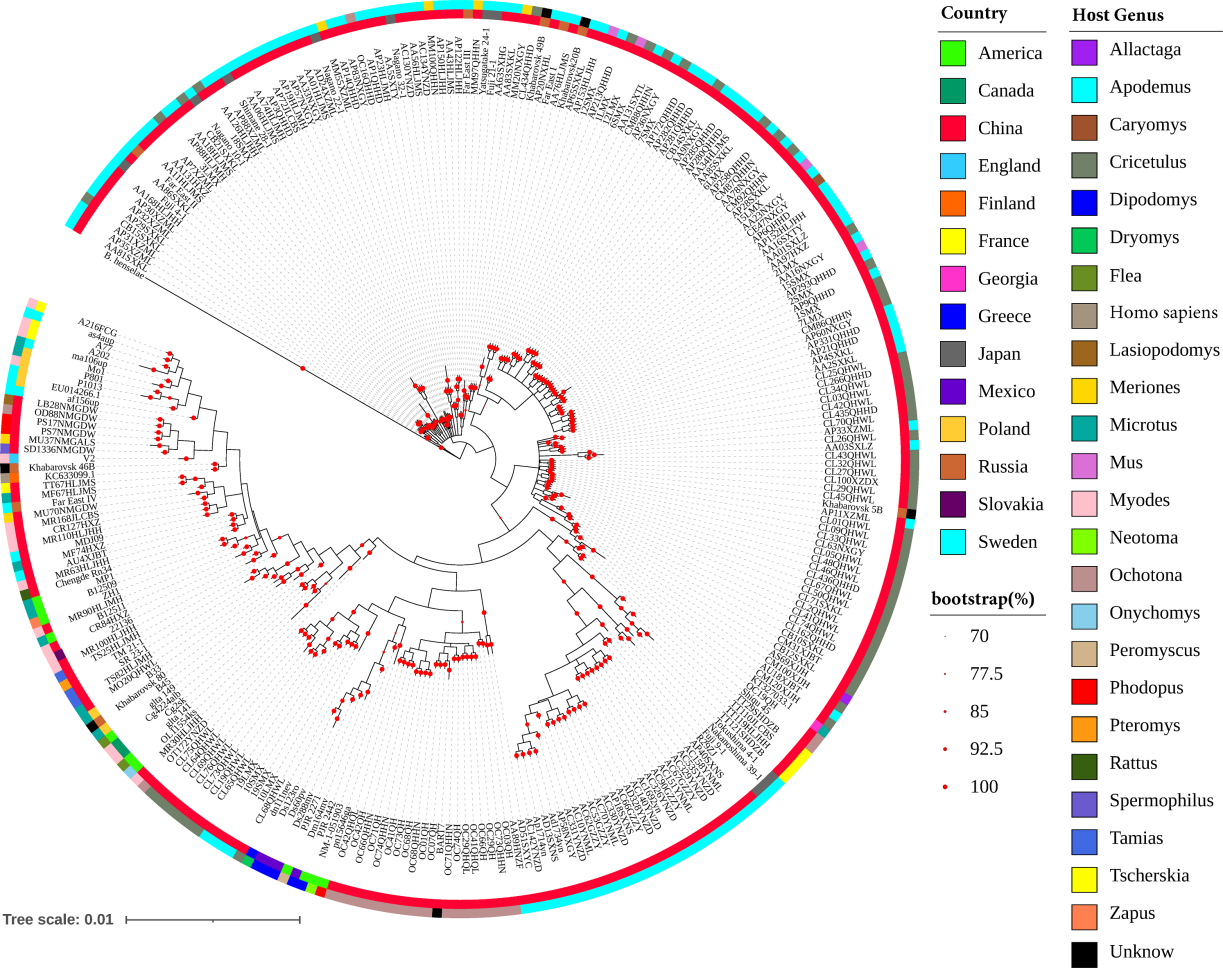
Phylogenetic tree based on 320 bp *gltA* genes of the 281 *B. grahamii* strains using MrBayes v3.2.7. *B. henselae* (NC_005956.1) was the outgroup. The different colors of the medial bands indicate *B. grahamii* country source, and the different colors of the lateral bands indicate the *B. grahamii* hosts.

The *gltA* gene alignment is commonly used to identify *Bartonella* species. To verify the relationship between 167 *B. grahamii* strains isolated in China and those isolated in other countries, the *gltA* gene was analyzed by phylogenetic analysis. Among the 281 *B. grahamii gltA* sequences, 136 were downloaded from the NCBI (Table S2). In the phylogenetic tree of the sequences (Fig. 1), *B. grahamii* isolated in China was found in each clade. In terms of the level of differentiation, the strains isolated from Shanxi and Tibet in China were located in the clade closer to the ancestor. The strains isolated in Russia and Japan were closely related to those isolated in China. *B. grahamii* (KT327033.1) isolated from Georgia was also clustered into a clade with the Chinese isolates. A202 (JX846173.1), A77 (JX846159.1), A216FCG (JQ694003.1) isolated from France and as4aup (CP001562.1) isolated from Sweden were a branch that diverged later. Based on the phylogenetic tree of the 323 bp *gltA* sequences, the global *B. grahamii* distribution showed a “mosaic pattern,” with some strains clustered according to geographical location. Similarly, at the host level, global *B. grahamii* showed a “mosaic distribution.” These results suggested that both host and geographical factors contributed to the evolution of *B. grahamii*. In this study, a comparative genomics study of *B. grahamii* was performed to further investigate the influence of host and geographic distribution on the genetic diversity of *B. grahamii*.

### Genomic features of *B. grahamii*

In this study, the assembled genome sequences of five *B. grahamii* isolated from Europe in the NCBI GenBank database were included in the analysis, including two sequences from the England, two from France, and one from Sweden (Table S3). The average nucleotide identity (ANI) values range of 172 strains, from 90.62% to 100%, were used to assess genomic similarity (Table S4). Recently, Amaral et al. noted in their study that the ANI for *B. vinsonii* group is 85%, and for *B. machadoae* group it is 91% (15). Based on the latest classification, we can more confidently assume that all strains, indeed, are represented by *B. grahamii*. The average genome size of 172 *B. grahamii* strains was 2.28 Mb (1.98–3.12 Mbp), the average number of contigs was 203, and the average N50 was 110.78 Kbp. According to Prokka annotation, the average number of coding sequences (CDS) was 1,911 (1,663–2,517), and the average number of rRNA, tRNA, and tmRNA was 4, 41, and 1, respectively. In addition, the average G + C content was 37.86% (37.44%–38.47%), and the average number of orthologous genes annotated by COG (clusters of orthologous groups) was 657 (610–772). The average number of hypothetical proteins was 968 (758–1,385).

A total of 247,909 variants were identified in 172 *B. grahamii* strains, including 164,761 SNPs and 83,148 Indels, using Snippy v4.6.0 and freebayes-parallel algorithms. VCFtools was used to screen SNPs with corresponding bases in all *B. grahamii* strains. After filtering, 35,870 (35,870/164,761) mutation sites were obtained to form a whole-genome SNPs data set of 172 *B. grahamii*. Using snpEff software for annotation, among the 35,870 SNPs, 27,509 (76.69%) were located in coding sequences (CDS) and 8,361 were located in intergenic regions. There were 19,793 synonymous mutations, 7,709 non-synonymous mutations, and 7 nonsense mutations in the coding region.

### Pangenome of *B. grahamii*

**Fig 2:**
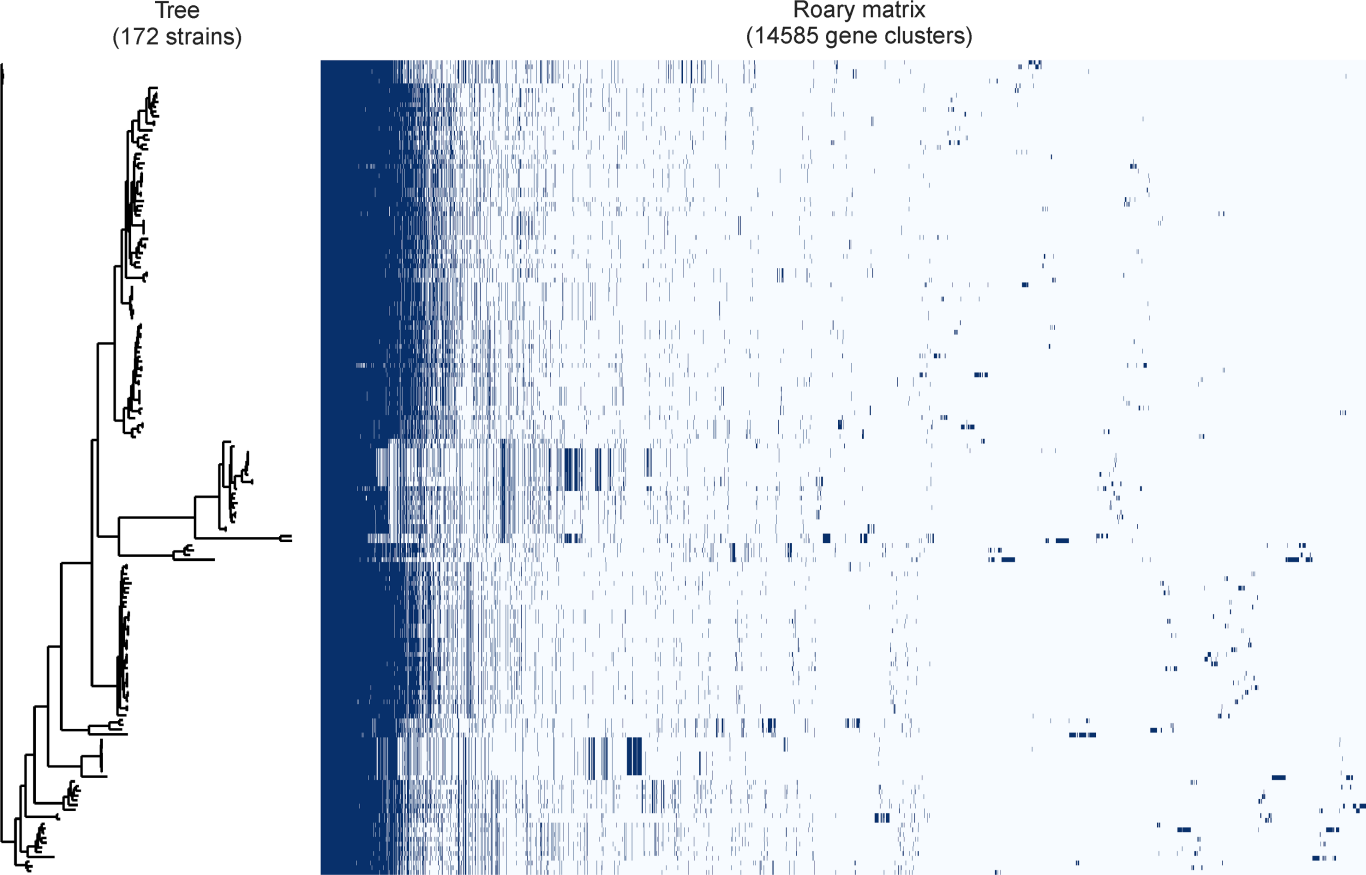
Pan-genome composition and distribution of 172 *B. grahamii* strains. The dendrogram on the left is the core-genome SNP-based phylogenetic tree that was constructed using IQ-TREE v1.6.12. Tree building was performed using the maximum likelihood (ML) method. Ultrafast bootstraps were calculated with 1,000 replicates. The gene distribution of each strain is shown on the right side of the figure.

*B. grahamii* genome assembly and annotation utilized the Prokka and Roary analyses. The total number of pan genes was 14,585, including 493 core genes (3.38%), 1,858 accessory genes (12.74%), and 12,234 cloud genes (83.88%). The phylogenetic tree constructed by the maximum likelihood method based on the core-genome SNP alignment sequences showed that 172 *B. grahamii* strains were divided into different branches, indicating that there was obvious genetic differentiation in 172 *B. grahamii* strains (Fig. 2).

According to Roary analysis, the number of core genes soon plateaued as the number of *B. grahamii* genomes increased, while the number of pan genes showed an increasing trend (Fig. S1A). As the number of genomes increased, new genes continued to be found at a fairly steady rate, and the number of unique genes discovered continued to increase (Fig. S1B). These trends indicated that the *B. grahamii* pan-genome was “open.”

Based on the core gene alignment file output by Roary software, a total of 30,626 polymorphic sites (S) and 25,527 parsimony information sites were detected in 172 *B. grahamii* strains. The number of gene haplotypes (h) was 144 (Table S1), the haplotype diversity (Hd) was 0.997, and the nucleotide diversity index (Pi) was 0.0257. These genetic diversity parameters indicated the high genetic diversity of *B. grahamii*.

In this study, the DNASP v5 was used to the neutrality test of 172 *B. grahamii* core genes. Tajima’s D (−0.67) and Fu and Li’s D (−0.08) were negative, indicating that the evolution pattern of *B. grahamii* was not consistent with neutral evolution. Using *B. grahamii* as4aup complete genome as the reference sequence, the average Ka/Ks ratio of *B. grahamii* was 0.86. This suggested that there was purifying selection during the *B. grahamii* evolution.

### Phylogenetic reconstruction of *B. grahamii*

**Fig 3:**
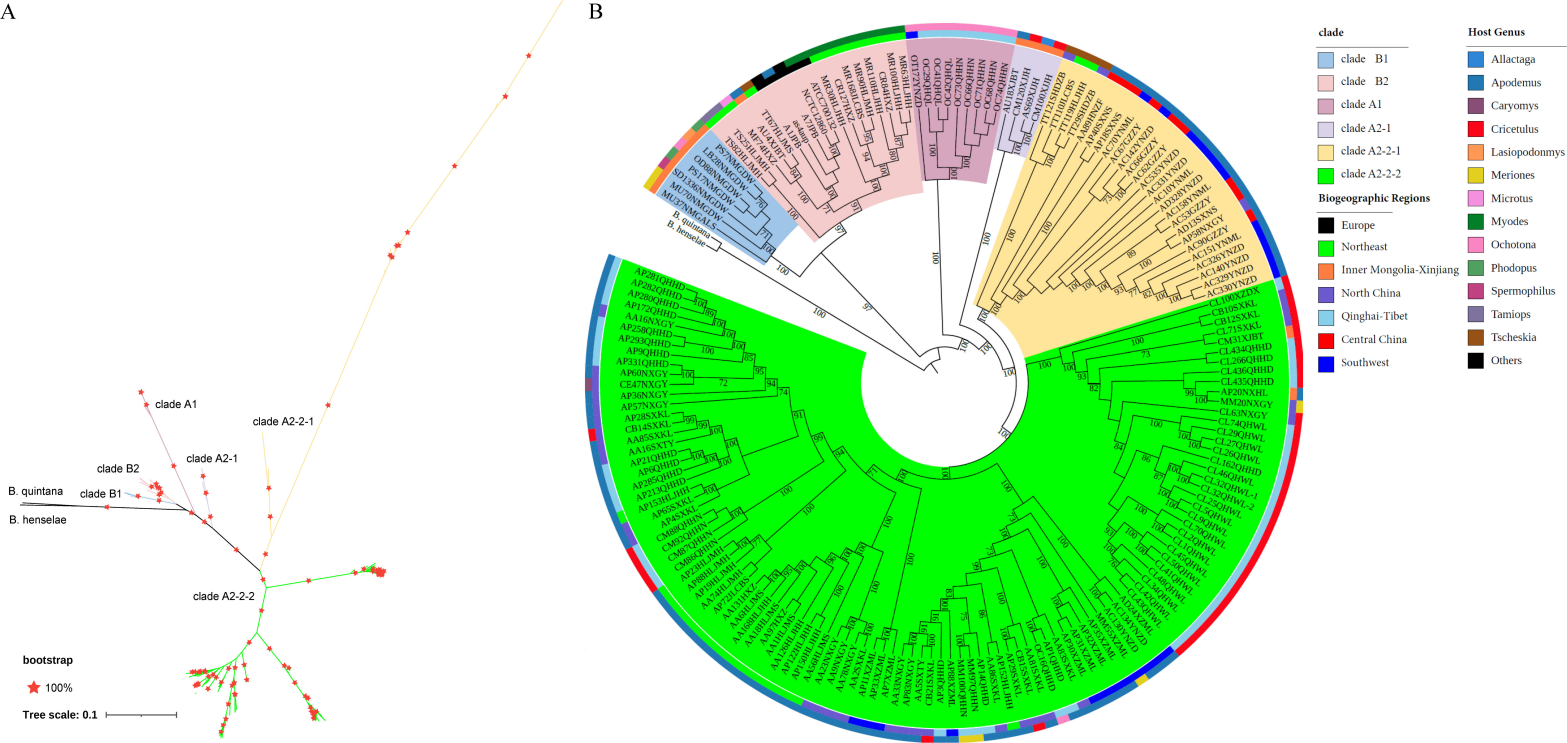
Phylogenetic analysis based on whole-genome SNPs of the 172 *B. grahamii* strains obtained by Snippy. *B. quintana* and *B. henselae* were used as outgroups. (A) The unrooted tree pattern of B. The color of the branches indicates clade grouping. (B) From the inside out, the label background colors indicate different clade, the inner bands indicate different biogeographic regions, and the outer bands indicate the *B. grahamii* isolated host.

The phylogenetic tree was constructed by the maximum likelihood method based on the high-quality SNPs data set (43,804 SNPs). According to the Bayesian information criterion (BIC), TVM + F + ASC + R3 was the best model. The 172 *B. grahamii* strains were divided into six clades (Fig. 3A). For biogeographic regions, clade B1 consisted of strains isolated from Inner Mongolia-Xinjiang region with different hosts. clade A2-1 was also composed of isolates from Inner Mongolia-Xinjiang region with different hosts. For rodent factor, nine strains from *Ochotona* belonged to clade A1, with strains isolated from southwest and Qinghai-Tibet regions. On the whole, *B. grahamii* formed mosaic aggregation according to the biogeographic regions of China and the host animals. The phylogenetic tree showed that the European strains shared a common ancestor with clade B2 Chinese strains and diverged later than some Chinese strains (Fig. 3B; Table S1).

### Principal component analysis and population structure analysis

**Fig 4:**
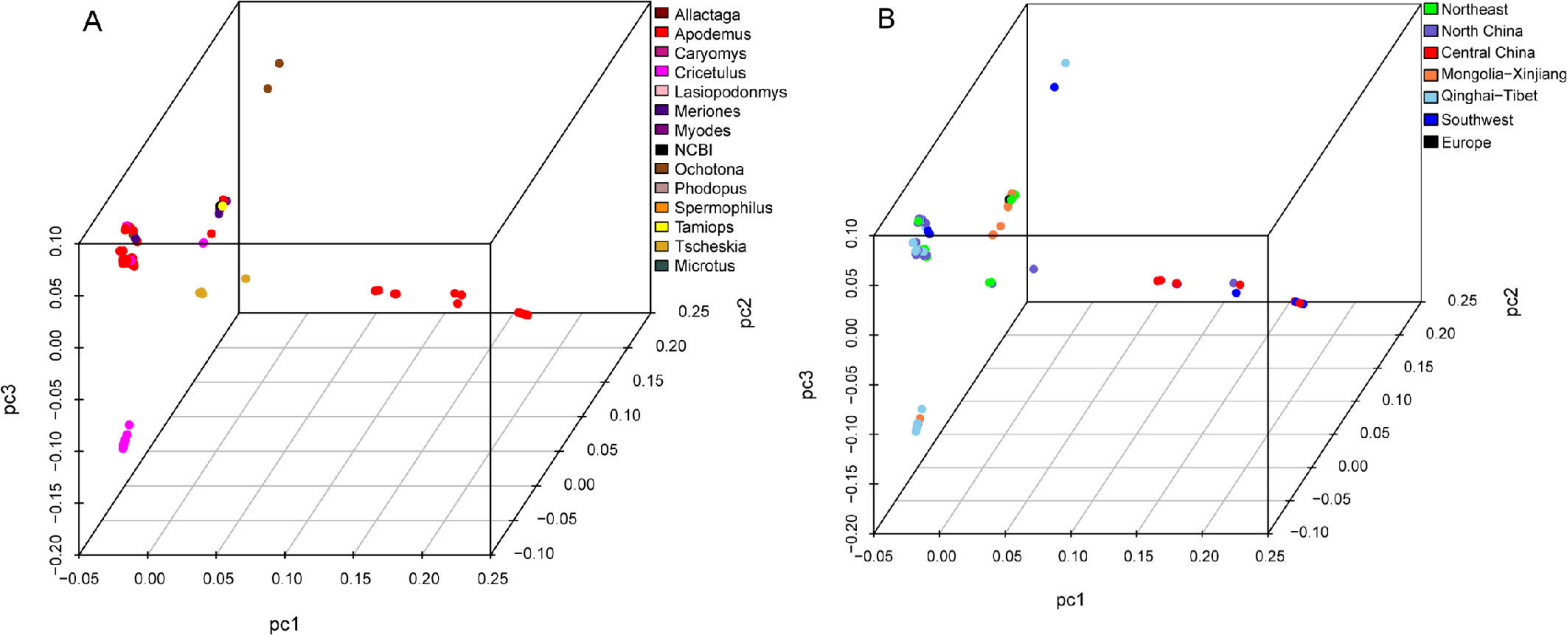
Principal component analysis of filtered whole-genome SNPs of the 172 *B. grahamii* strains. (A) Different colors indicate different host animals. (B) Different colors indicate different biogeographic regions.

PCA analysis was performed on a high-quality genome-wide SNPs (35,870 SNPs) data set of *B. grahamii*. Strains isolated from the same genus of rodents were more likely to cluster together than strains isolated from the same biogeographic regions (Fig. 4).

**Fig 5:**
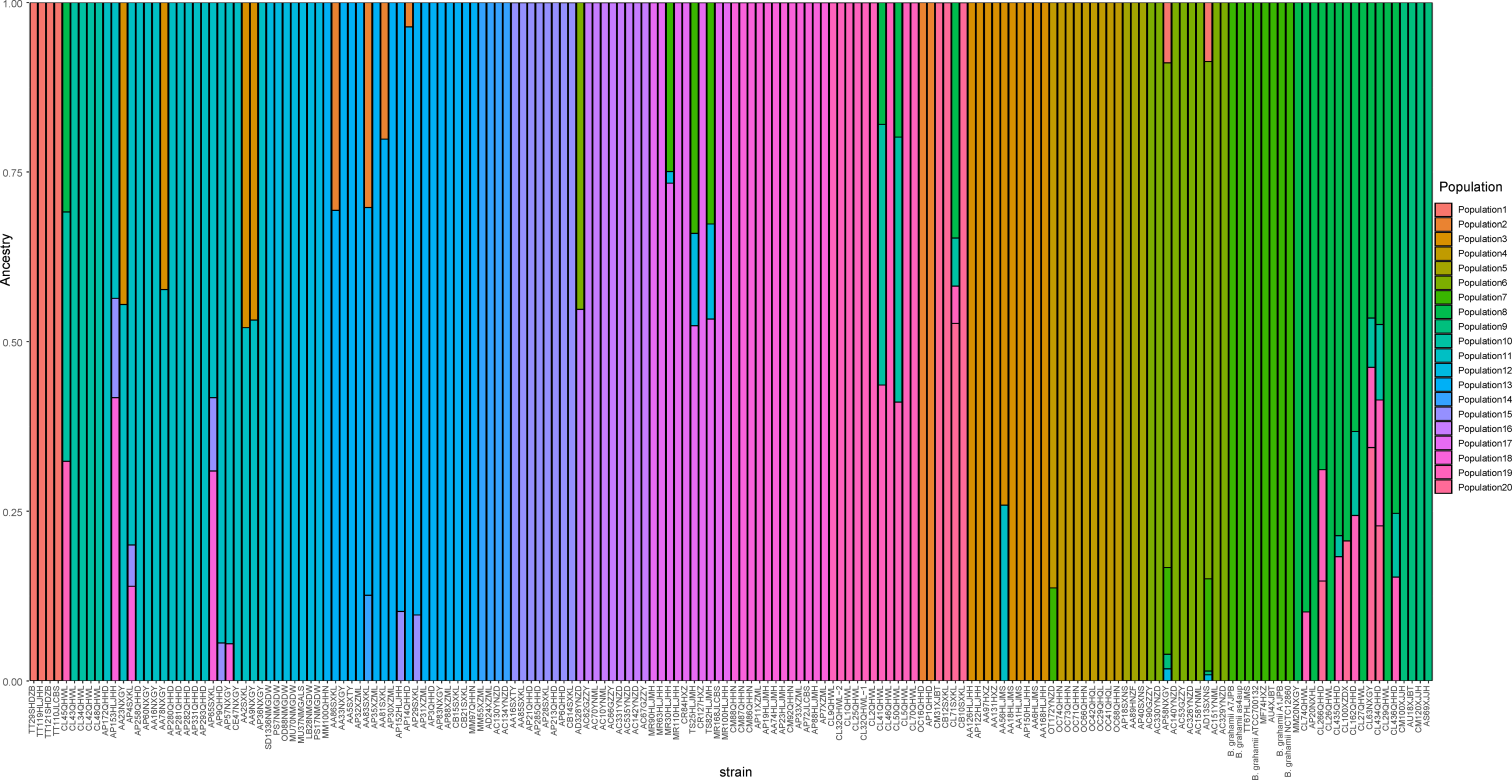
Population genomics analysis of 172 *B. grahamii* strains based on 35,870 SNP loci. The *y*-axis is the population membership, and the *x*-axis is the strain. Each vertical bar represents a sample, and different colors represent separate populations (*K* = 20).

After filtering with VCFtools and PLINK software, 35,870 loci were used for the analyses of genetic structure and diversity. By calculating and comparing the minimum CVe values, the best *K* value was obtained to be 20. The ADMIXTURE analysis to access the genomic population structure of 172 samples of *B. grahamii* based on 35,870 SNPs suggested the existence of 20 genetic groups (Fig. 5; Table S1) based on the *K*. Five well-defined subgroups (population 1, 4, 6, 16, and 20) strongly associated with the host origin of the samples were evident. Population 1 was composed of 4 strains from *Tscherskia* from Northeast and North China (Shandong, Heilongjiang, and Jilin). Population 4 comprised 9 *B. grahamii* from *Ochotona*. Among them, one sample (OT172YNZD) was collected in Southwest and eight in Qinghai-Tibet. The strain (OT172YNZD) from Yunnan showed a low mixture level with population 7. Population 6 consisted of 10 strains from *Apodemus*. Population 16 was formed by nine strains isolated from *Apodemus*. Population 20 exclusively consisted of four strains from *Cricetulus*, including one from Inner Mongolia-Xinjiang and three from North China.

There were five subgroups (populations 2, 9, 12, 14, and 17) closely related to the origin of the biogeographic regions of the samples (Fig. 5; Table S1). Population 2 consisted of two strains collected in Qinghai-Tibet, which were isolated from tissues of *Ochotona* and *Apodemus*, respectively. Population 9 consisted of four strains isolated from the Inner Mongolia-Xinjiang region (Xinjiang Uygur Autonomous Region), China. Population 12 was also composed of seven *B. grahamii* strains from the Inner Mongolia-Xinjiang region (Inner Mongolia). Four strains isolated from Southwest China constituted population 14. Population 17 consisted of 10 strains isolated from Northeast China. Of these, eight strains were isolated from tissues of *Myodes* and two strains were isolated from tissues of *Tamias*.

Four subgroups (population 3, 5, 10, and 19) were closely related to both the host and biogeographic regions of the samples (Fig. 5; Table S1). Population 3 was composed of 10 strains from *Apodemus* collected in the Northeast region. Population 5 consisted of 3 strains isolated from Central China, also obtained from tissues of *Apodemus*. Five *B. grahamii* strains isolated from *Cricetulus* tissue samples collected in the Qinghai-Tibet region comprised population 10. Population 19 was also composed of 11 strains of *B. grahamii* isolated from *Cricetulus* collected in Qinghai-Tibet.

### Population structure variation

Samples were grouped based on host genus and biogeographic regions in China to analyze the molecular variance of the core genome of 172 *B. grahamii*. When grouped by host genus, the variation among populations accounted for 43.63% of the total variation, and the variation within each population accounted for 56.37% (Table 1). This suggested genetic differentiation of *B. grahamii* populations isolated from different genera of rodents. In addition, there were other factors contributing to *B. grahamii* variation, which might include geographical factors.

**TABLE 1:** AMOVA among *B. grahamii* populations in different genera rodents

Source of variation	d.f.	Sum of squares	Variance components	Percentage of variation	Fixation index	*P* value
Among populations	13	158,202.49	1,201.20Va	43.63		
Within populations	158	245,215.00	1,551.99Vb	56.37		
Total	171	403,417.49	2,753.20		0.44	0.00

According to the grouping results of biogeographic regions, the variation among populations accounted for 33.49%, and the variation within populations accounted for 66.52% (Table 2). This indicated that biogeographic regions play a role in the differentiation of *B. grahamii*, but the role was less than that of host factors. The correlation analysis between isolation sources and SNP sequences of 172 *B. grahamii* isolates obtained the same results at the genome-wide scale (Fig. S2). Hosts of isolates and genome-wide SNPs association analysis showed that approximately 90 SNPs were significantly associated with hosts (Fig. S2A). Approximately 30 SNPs were associated with the biogeography of isolates, one-third of SNPs associated with the hosts of isolates (Fig. S2B).

**TABLE 2:** AMOVA among *B. grahamii* populations in different biogeographic regions

Source of variation	d.f.	Sum of squares	Variance components	Percentage of variation	Fixation index	*P* value
Among populations	6	125,910.62	846.40Va	33.48		
Within populations	165	277,506.87	1,681.86Vb	66.52		
Total	171	403,417.49	2,528.26		0.33	0.00

**Fig 6:**
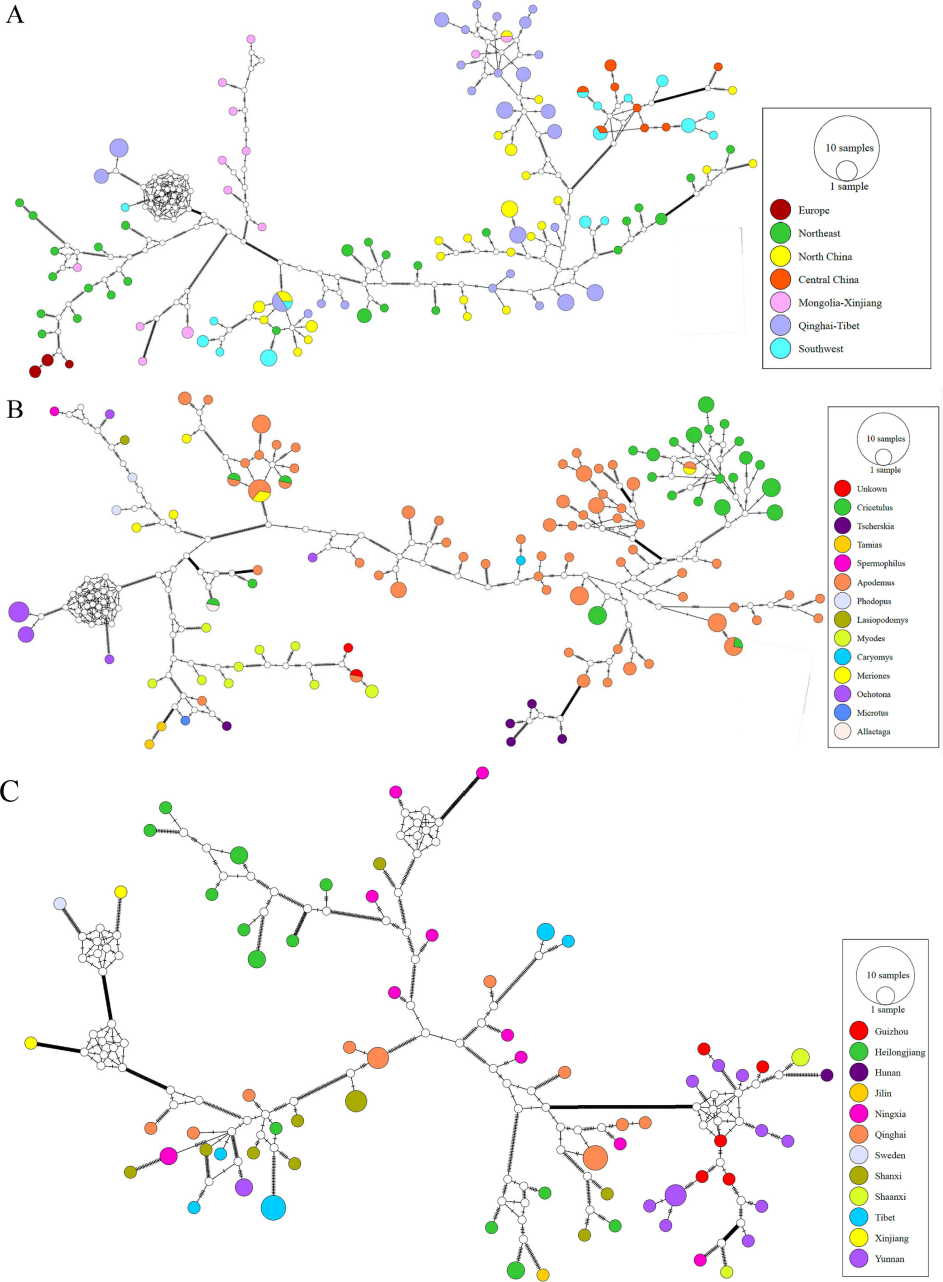
Median-joining network inferred from *B. grahamii* core genome. Each colored circle represented a haplotype and circle size indicated the number of samples. Each color represented a sampling area. The line between two circles indicated that two haplotypes were related to each other. Short lines along the lines indicated the number of base substitutions required between haplotypes. Each hollow circle represented the inferred haplotype by the PopART software without an actual sample. (A and B) Network of *B. grahamii* haplotype diversity obtained from 172 samples China and Europe. (A) Each color represented a biogeographic region. (B) Each color represented a host genus. “Unkown” meant that there was no clear host genus in NCBI. (C) The phylogenetic network for the core genomes of the 89 *B. grahamii* strains constructed according to the rodent host’s genus taxonomic level *Apodemus* spp.. Each color represented a sampling area.

To investigate the distribution of *B. grahamii* on a population level across host and geography isolates, we employed a network of haplotype diversity analysis to reveal the genetic structure and evolutionary relationships between different populations. First, to eliminate the impact of recombination, we removed recombinant genes from the core gene alignment sequence using Gubbins (16). Network analysis indicated a remarkable correlation between core genome and host genera. Although core genomes of *B. grahamii* obtained from *Apodemus*—and from *Cricetulus*, *Tscherskia* and *Myodes*—tended to cluster together, there was microevolution in the same host genus (Fig. 6). We noted the existence of a small number of clusters made of isolates from multiple host genus. These results suggested that the genetic differentiation of *B. grahamii* was influenced not only by the host genus but also by other factors. Network graph of core genome between isolates from *Apodemus* indicated strains from Hunan, Guizhou, Shaanxi, Ningxia, and Yunnan had a common ancestor. *B. grahamii* had a radiation trend from central China to northwest China and had microevolution under the effect of geographical barriers (Fig. 6).

## DISCUSSION

In this study, the genome sequences of 167 wild *B. grahamii* strains in China and 5 *B. grahamii* strains published in NCBI were analyzed. A total of 167 wild strains were sampled in 13 provinces and distributed in 6 biogeographic regions of China. These results indicated that *B. grahamii* wild strains had strong adaptability to environment and host. This survey provided great value for the epidemiological study of *B. grahamii* in China.

The phylogenetic tree based on 323 bp *gltA* gene fragments showed that both host and geographic factors played a role in the genetic differentiation of *B. grahamii*. Previous studies have shown that phylogenetic trees based on the gene fragments are not completely in line with those based on genome-wide SNPs (17, 18). Therefore, this study explored the roles of rodents and biogeographic regions on the genetic differentiation of *B. grahamii* at genome level. The genome size of *B. grahamii* with relatively complete assembly (number of contigs < 200) ranged from 1.98 to 2.39 Mbp, with an average of 2.22 Mbp. *B. grahamii* genome was larger than that of *B. quintana* (1.5–1.7 Mbp) and *B. henselae* (about 2 Mbp). These suggested that there were high horizontal gene transfer and gene duplication in *B. grahamii* genomes (19), which might be a reason why *B. grahamii* coped well with different survival environments. A total of 247,909 mutations were identified in the 172 *B. grahamii* by Snippy. After filtering, 35,870 high-quality SNP sites were used for annotation. Annotation results showed that 76.69% of mutation sites occurred in the coding region, of which 28.02% (7,709/27,509) were non-synonymous mutations. This indicated that *B. grahamii* genomes had high genetic diversity and strong plasticity. Through pan-genome analysis, the proportion of core genes (3.38%) in *B. grahamii* was small, and the proportion of specific genes (83.88%) was relatively high. This indicated that *B. grahamii* had a highly diverse gene pool to adapt to different hosts and various living environment.

In order to clarify the population structure and genetic differentiation of *B. grahamii*, we analyzed the population genetic structure of 172 *B. grahamii* strains based on genome-wide SNPs data set. The phylogenetic tree based on genome-wide SNPs of *B. grahamii*, with *B. henselae* and *B. quintana* as outgroups, revealed a “mosaic distribution” in whole *B. grahamii* population (Fig. 3). The European isolates diverged later than some of the Chinese isolates and shared a common ancestor with the wild Chinese isolates. According to this result and previous study, the European strains might be the product of genetic differentiation of the Asian strains (14). However, only five European *B. grahamii* genomes were obtained in this study. More samples are needed to further verify the evolutionary relationship between Europe and Asia *B. grahamii* in the future.

To further understand internal clustering and gene exchange of *B. grahamii* clade, the population structure of 172 *B. grahamii* was analyzed by ADMIXTURE. *B. grahamii* strains were divided into 20 populations (Fig. 5). Five European isolates together with those from Northeast and the Inner Mongolia-Xinjiang region in China constituted in population 7. This again suggested that European strains in this study might originate from Asia. Population structure analysis showed that *B. grahamii* clustered by both hosts and biogeographic regions. Some populations (population 3, 5, 10, and 19) were composed of strains isolated from the same host in the same region. According to the molecular variance analysis, there was 43.63% variation among populations grouped by host and 33.48% variation among populations grouped by biogeographic regions (Tables 1 and 2,). These results suggested that host factors might play a more important role in the genetic differentiation of *B. grahamii*. In both classifications, individual differences within groups were predominant, indicating the complexity and diversity of *B. grahamii* genomes. Haplotype networks (Fig. 6) showed that strains isolated from *Apodemus* spp. had microevolution in some regions, which might be due to terrain factors such as yunnan-guizhou plateau. This corresponded to “mosaic pattern” distribution and suggested that genetic differentiation of *B. grahamii* was also influenced by geographic isolation. In the study of the evolutionary history of field mice, Ge et al. mentioned that the terrain of China is a complex mosaic, and this habitat heterogeneity seems to favor the diversification of *Apodemus* species (20). The differentiation of *Apodemus* species might be a further cause leading to the microevolution of *B. grahamii*, which separated from this genus. This again suggested a co-evolutionary relationship between *B. grahamii* and hosts.

In this study, we established a genomic data set of 167 *B. grahamii* strains isolated from China, which added a large amount of Chinese data to the study of *B. grahamii*. The pan-genome and genome-wide characteristics of *B. grahamii* were systematically studied by various methods, and the genetic differentiation and gene exchange of *B. grahamii* were studied, which provided scientific basis for accurate classification and tracing of *B. grahamii*.

## MATERIALS AND METHODS

### *B. grahamii* collection and DNA extraction

Our research group engages in the detection and isolation of *Bartonella* species in China. Under sterile conditions, approximately 10 mg of organ samples (liver or spleen) were added to a sterilized 2 mL centrifuge tube (containing 100 µL tryptic soy broth and some 1.2 mm grinding beads). After homogenization with a tissue grinder, the suspensions were inoculated onto tryptic soy agar (TSA) plates containing 5% defibrinated sheep blood and cultured at 36.5℃ for 20 days in a humidified incubator with 5% CO_2_. Next, suspected *Bartonella* colonies were picked for purification, separation, and subculture 2–4 times. The pure bacterial culture was collected and stored at −80℃ in brain heart infusion broth containing 30% glycerol. Collected strains with ≥97% (21) similarity to the full-length *gltA* sequence of *B. grahamii* were selected for recovery and nucleic acid extraction. The Wizard Genomic DNA purification Kit (Promega, Madison, WI, USA) was used to extract genomic DNA from the selected strains according to the manufacturer’s instructions. The nucleic acid concentration was determined by spectrophotometer, and samples with nucleic acid concentration at least 50 ng/µL were used for genome sequencing.

### Sequencing of *B. grahamii* genome

The DNA samples qualified by electrophoresis were randomly divided into 350 bp fragments by a Covaris ultrasonic crusher to construct a small fragment library. Paired-end sequencing was performed on the Illumina PE150 platform with an average sequencing depth of about 100×. Readfq (v10) was used to perform quality control on raw data and filter low-coverage sequences to obtain clean data. *De novo* assembly was performed in strains using SOAP denovo v2.04 (22), SPAdes v3.13.0 (23), and ABySS (24). Prokka v1.14.5 (25) was used to annotate *B. grahamii*, including re-annotation of the genome sequences downloaded from NCBI (Table S3), using default parameters. Quast v5.2.0 (26) evaluated the genome assembly results and counted the general information of the genome.

### Phylogenetic analysis of the *gltA*

To explore phylogenetic relationships of global *B. grahamii*, we used sequences downloaded from NCBI with ≥97% identity to the *gltA* gene fragment of *B. grahamii* to perform phylogenetic analysis, with *B. henselae* as an outgroup (Table S2). Gene sequences were aligned by using ClustalW. Haplotype networks using software PopART v.1.7 (27). A phylogenetic tree was constructed by means of Bayesian Markov Chain Monte Carlo (MCMC) inference using MrBayes v3.2.7 (28, 29). Applying the Akaike information criterion (AIC), Modeltest v3.7 assigned to the Lset and Prset parameters (30, 31). Four chains of the MCMC were run simultaneously and sampled every 100 generations for a total of 16 million generations. Trees were visualized with iTOL online software (https://itol.embl.de/).

### Pan-genome and core-genome analysis

Pairwise average nucleotide identity (ANI) values were calculated using FastANI v1.33 (32) to determine the real identity of isolates at the genomic level. The resultant gff3 files by Prokka were used for pan-genome analysis with Roary v3.13.0 (33, 34). The minimum identity with BLASTp was set to 95%. Core genes were extracted using TBtools software (https://github.com/CJ-Chen/TBtools) combined with a gene presence/absence matrix generated by Roary. Single-nucleotide polymorphisms (SNPs) were aligned with MAFFT v7.490 (35) and were later used for phylogenetic analyses.

DnaSP v6 (DNA sequence polymorphism) was used to calculate the haploype diversity (Hd), nucleotide diversity (Pi), and Tajima’s Test of the core gene alignment file generated by Roary software diversity Pi).

### Genome-wide SNPs calling and phylogeny construction

SNPs were called using Snippy v4.6.0 with strain as4aup (GenBank accession no. CP001562) used as reference. Recombination was filtered using Gubbins v2.4.1 (16). Next, VCFtools v0.1.13 (36) was employed to obtain high-quality SNPs with the parameters “--max-missing 1 --min-alleles 2 --max-alleles 2—maf 0.05.” To ensure the independence of nearby SNPS in subsequent analyses, PLINK v1.9 (37) was used for SNP pruning based on linkage disequilibrium (LD), with “--indep-pairwise 1000 10 0.2.” The filtered genome-wide SNPs were annotated using snpEff (38) software.

Maximum likelihood (ML) phylogenetic trees using high-quality SNPs were generated by IQ-TREE v1.6.12 (39). The best model was selected by the ModelFinder (40) program. The number of ultrafast bootstrap replicates was 1,000. Trees were visualized on iTOL website (https://itol.embl.de/).

### Population structure and PCA analysis

PLINK v1.9 was used to convert files of high-quality SNPs into plink data set files. Using filtered SNP data, ADMIXTURE v1.3.0 was used to reconstruct population structure and estimate the admixture proportions among *B. grahamii* populations. Six scenarios, ranging from *K* = 2 to *K* = 30, were selected for genetic clustering. The optimal number of groups was identified based on the *K* value with the lowest fivefold cross-validation error (CVe). The principal component analysis (PCA) was performed in PLINK v1.9 using the “–pca 20” parameter and calculated the top 20 PCs. The results of ADMIXTURE and PCA were plotted using R packages.

Next, we analyzed the association between genome-wide SNPs of *B. grahamii* and host origins and biogeographic regions of isolates by GEMMA v0.98.5 software (41). Filtered SNP data were used as GEMMA input file to generate the standardized matrix. GWAS analyses were performed using mixed linear models with the Wald test. An overview of the test results was shown as Q-Q plots and Manhattan plots constructed by the statistical package R.

### Analysis of molecular variance

Population grouping of individual sequences was performed using DnaSP v6 software based on the core gene alignment file obtained by Roary software and saved as Arlequin file format. Arlequin v3.5 (42) software was used for analysis of molecular variance (AMOVA). All populations were divided into one group in Arlequin software.

### Downloading publicly available genome sequences

The assembly genome sequences of validly named *B. grahamii* were downloaded from the NCBI public database on 1 May 2023. Table S3 shows *B. grahamii* downloaded from the NCBI that were included in genomic analysis.

## Data Availability

The *B. grahamii* genome sequences have been deposited in the GenBank database under BioSample accession numbers SAMN32552675, SAMN32580103, SAMN32580104, and SAMN43214729–SAMN43214892.

## SUPPLEMENTAL MATERIAL

Fig. S1 msystems.01089-24-s0001.tif Characteristic curves of number of genes in 172 strains of *B. grahamii*.

Fig. S2 msystems.01089-24-s0002.tif Q-Q plots and Manhattan plots of GEMMA results for 172 *B. grahamii* isolations.

Table S1 msystems.01089-24-s0003.xlsx General background information and genetic classification of *B. grahamii* isolations in China.

Table S2 msystems.01089-24-s0004.xlsx *B. grahamii* from NCBI selected for the phylogenetic tree.

Table S3 msystems.01089-24-s0005.xlsx General information of *B. grahamii* downloaded from NCBI.

Table S4 msystems.01089-24-s0006.xlsx ANI between isolations.

ASM does not own the copyrights to Supplemental Material that may be linked to, or accessed through, an article. The authors have granted ASM a non-exclusive, world-wide license to publish the Supplemental Material files. Please contact the corresponding author directly for reuse.
