# Supplementary figures and images for: Microevolution of Bartonella grahamii driven by geographic and host factors
Source: mSystems. 2024 Sep 30;9(10):e01089-24. doi: 10.1128/msystems.01089-24 (PMC11494883; doi:10.1128/msystems.01089-24)

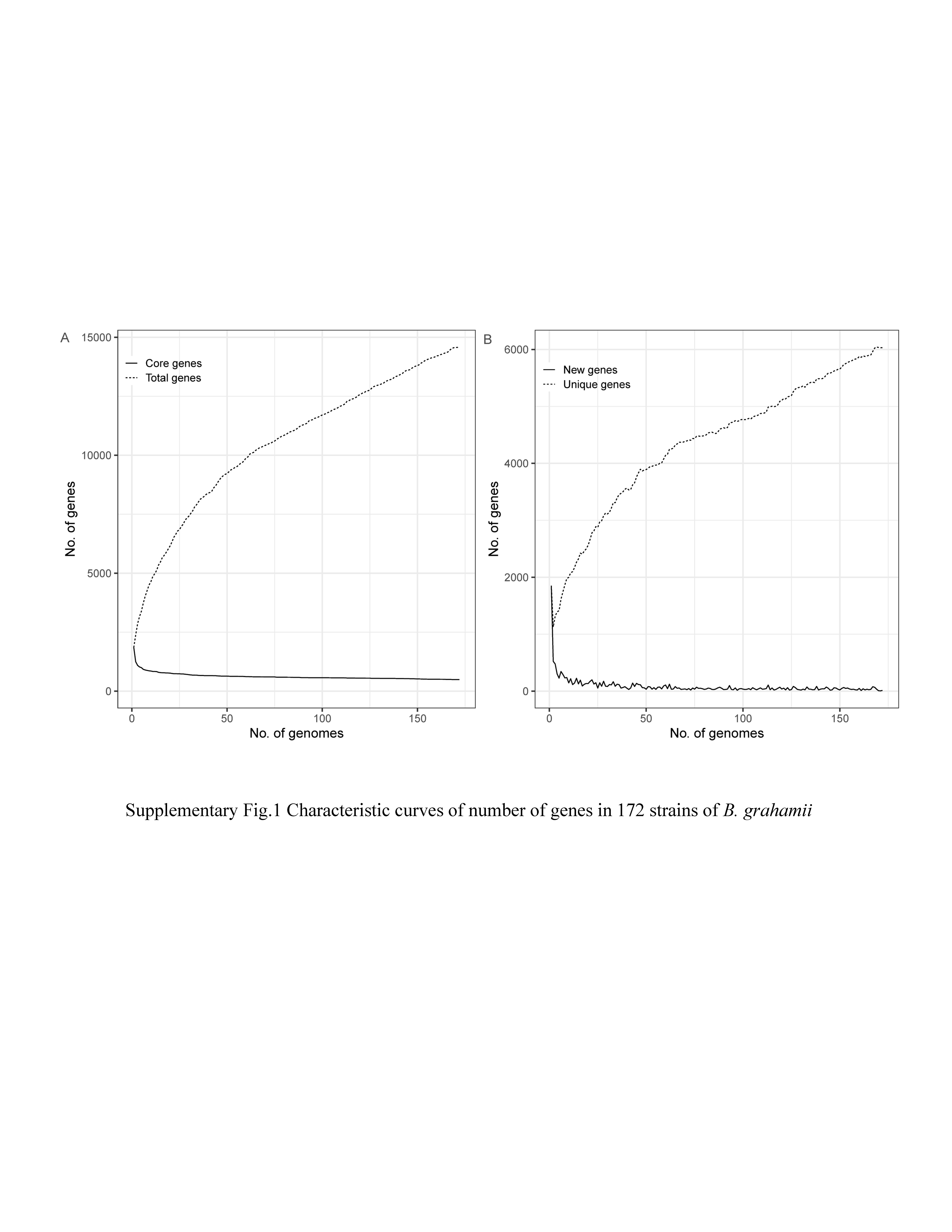

Supplement: Fig. S1 — Characteristic curves of number of genes in 172 strains of B. grahamii. [file msystems.01089-24-s0001.tif]

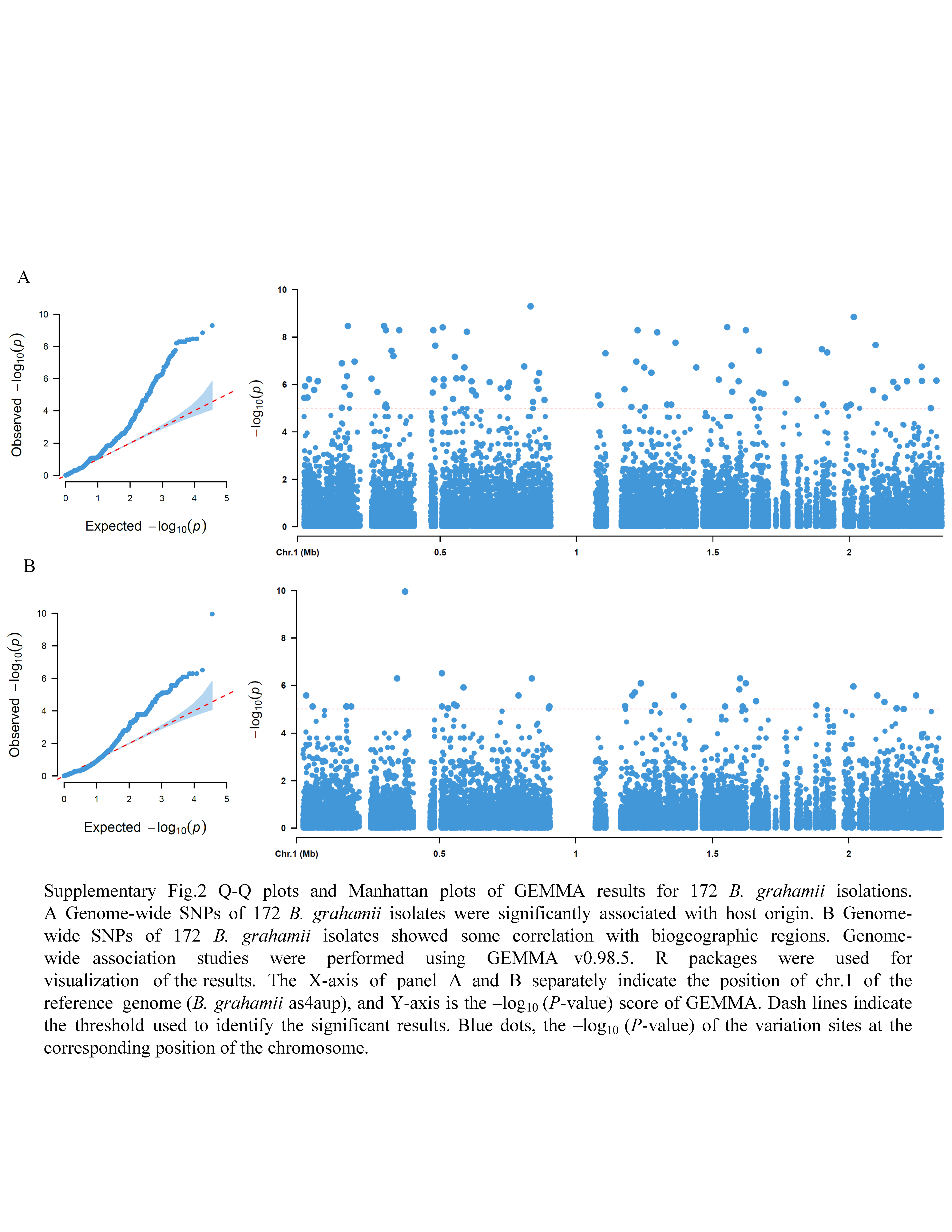

Supplement: Fig. S2 — Q-Q plots and Manhattan plots of GEMMA results for 172 B. grahamii isolations. [file msystems.01089-24-s0002.tif]
